# Supplementary material for: Impact of pain on functional status and quality of life in Jordanian women with breast cancer
Source: PLoS One. 2024 Oct 16;19(10):e0307271. doi: 10.1371/journal.pone.0307271 (PMC11482730; doi:10.1371/journal.pone.0307271)
Supplement: Attachment — Submitted filename: Response to Reviewers.docx [file pone.0307271.s001.docx]

**Response to Reviewers' Comments**

| **No** | **Comments from the**  **Reviewers** | **Response** | **Reflected on** |  |
| --- | --- | --- | --- | --- |
|  | **General:**  **Journal Requirements** |  |  |  |
| **1.** | Please ensure that your manuscript meets PLOS ONE's style requirements | The manuscript was revised to meet PLOS ONE's style requirements | *All over the manuscript* | ^<https://www.google.com/imgres?imgurl=http://www.maltaxterra.com/wp-content/uploads/2015/03/big_tick.png&imgrefurl=http://www.maltaxterra.com/race-entry-list-end-february/&docid=BiOg4ILiGrs_mM&tbnid=bF9PzfQ9lblDFM:&vet=1&w=256&h=256&bih=652&biw=1366&ved=0ahUKEwiW9pvutI3XAhUMEVAKHYoxAzsQxiAIHSgF&iact=c&ictx=1>^ |
| **2.** | Please confirm at this time whether or not your submission contains all raw data required to replicate the results of your study. Authors must share the "minimal data set" for their submission. | Checked and revised accordingly | *All over the manuscript* | ^<https://www.google.com/imgres?imgurl=http://www.maltaxterra.com/wp-content/uploads/2015/03/big_tick.png&imgrefurl=http://www.maltaxterra.com/race-entry-list-end-february/&docid=BiOg4ILiGrs_mM&tbnid=bF9PzfQ9lblDFM:&vet=1&w=256&h=256&bih=652&biw=1366&ved=0ahUKEwiW9pvutI3XAhUMEVAKHYoxAzsQxiAIHSgF&iact=c&ictx=1>^ |
| **3.** | Please include your full ethics statement in the 'Methods' section of your manuscript file. | Revised and rewritten | ***Line 178-181***  Ethical consideration under the Materials and Methods section  Two sentences add  *The survey methodology ensured maintaining confidentiality; patients were informed of anonymity and confidentiality. No known personally identifiable information was gathered. The responses were saved in the principal investigator's personal computers, secured with passwords.* | ^<https://www.google.com/imgres?imgurl=http://www.maltaxterra.com/wp-content/uploads/2015/03/big_tick.png&imgrefurl=http://www.maltaxterra.com/race-entry-list-end-february/&docid=BiOg4ILiGrs_mM&tbnid=bF9PzfQ9lblDFM:&vet=1&w=256&h=256&bih=652&biw=1366&ved=0ahUKEwiW9pvutI3XAhUMEVAKHYoxAzsQxiAIHSgF&iact=c&ictx=1>^ |
| **4.** | Please upload a copy of Supporting Information Table. Supporting information which you refer to in your text on page 23-24. | Uploaded | *Tables 1-13* | ^<https://www.google.com/imgres?imgurl=http://www.maltaxterra.com/wp-content/uploads/2015/03/big_tick.png&imgrefurl=http://www.maltaxterra.com/race-entry-list-end-february/&docid=BiOg4ILiGrs_mM&tbnid=bF9PzfQ9lblDFM:&vet=1&w=256&h=256&bih=652&biw=1366&ved=0ahUKEwiW9pvutI3XAhUMEVAKHYoxAzsQxiAIHSgF&iact=c&ictx=1>^ |
| **5.** | Please review your reference list to ensure that it is complete and correct. | Revised and corrected where it is required | *References list* | ^<https://www.google.com/imgres?imgurl=http://www.maltaxterra.com/wp-content/uploads/2015/03/big_tick.png&imgrefurl=http://www.maltaxterra.com/race-entry-list-end-february/&docid=BiOg4ILiGrs_mM&tbnid=bF9PzfQ9lblDFM:&vet=1&w=256&h=256&bih=652&biw=1366&ved=0ahUKEwiW9pvutI3XAhUMEVAKHYoxAzsQxiAIHSgF&iact=c&ictx=1>^ |
|  | **Reviewer #1:** |  |  |  |
| **1.** | All abbreviations should be spelled out when they appear for the first time in the text or even in the tables. | I agree, Corrected | ***For example, Lines 201-210***  *Physical health-related domains include General Health (GH), Physical Functioning (PF), Role Physical (RP), and Body Pain (BP). The overall score of this questionnaire is given by two scores: a mental component scale (MCS) and a physical component scale (PCS).* | ^<https://www.google.com/imgres?imgurl=http://www.maltaxterra.com/wp-content/uploads/2015/03/big_tick.png&imgrefurl=http://www.maltaxterra.com/race-entry-list-end-february/&docid=BiOg4ILiGrs_mM&tbnid=bF9PzfQ9lblDFM:&vet=1&w=256&h=256&bih=652&biw=1366&ved=0ahUKEwiW9pvutI3XAhUMEVAKHYoxAzsQxiAIHSgF&iact=c&ictx=1>^ |
| **2.**  **2.1.**  **2.2.**  **2.3.**  **2.4.** | Some editing is needed. For example, on page 5 line 119, instead of saying "to hit the light," it should say to shed the light.  On line 120, an extra period should be deleted after the word enhancement.  also page 7, the spelling of the months is not correct (line 156).  On page 8, line 182, 4 concepts, not eight, or mention the eight concepts if they are 8.  On page 11, line 228, it should read "with a range," not "arrange." | Changed  Removed  Corrected  The section is rewritten to be precise.  Corrected | ***Line 130***  *It is time to shed light on further research targeting life improvement and functional status enhancement.*  ***Line 175***  *Data were collected From August 1^st,^ 2022, to November 5^th,^ 2022.*  ***For example, Lines 201-210***  *The SF-12 is a shorter version of the SF-36 questionnaire. The SF-12 is a health-related quality-of-life questionnaire consisting of twelve questions that measure eight health domains to assess physical and mental health. Physical health-related domains include General Health (GH), Physical Functioning (PF), Role Physical (RP), and Body Pain (BP). The overall score of this questionnaire is given by two scores: a mental component scale (MCS) and a physical component scale (PCS). A score of 50 or less on the PCS-12 has been recommended as a baseline for identifying a physical condition, while a score of 42 or less on the MCS-12 may indicate 'clinical depression.' The Arabic version has been tested for reliability and validity in a study in Lebanon [20]. For this study, Cronbach's alpha for mental and physical components is 0.760, 0.703.*  ***Line 263***  *whereas the physical component subscale was 38.6 (SD=5.1), with a range between 26.68 and 53.86.* | ^<https://www.google.com/imgres?imgurl=http://www.maltaxterra.com/wp-content/uploads/2015/03/big_tick.png&imgrefurl=http://www.maltaxterra.com/race-entry-list-end-february/&docid=BiOg4ILiGrs_mM&tbnid=bF9PzfQ9lblDFM:&vet=1&w=256&h=256&bih=652&biw=1366&ved=0ahUKEwiW9pvutI3XAhUMEVAKHYoxAzsQxiAIHSgF&iact=c&ictx=1>^ |
| **3.**  **3.1.**  **3.2.** | Additionally, lines 282-285,  the paragraph on page 14, and the text lines 276-279,  276-291, require editing. | Absolutely yes  Rewritten to be clear  Rewritten to be clear  Rewritten to be clear | ***Line 315-326***  *In addition to the correlation analysis, a hierarchical linear regression analysis was conducted to measure the impact of pain severity (independent variable) on the quality of life of cancer patients (dependent variable) while considering the influence of cancer stage. Furthermore, this analysis was employed to manage other factors, particularly the cancer stage effectively.*  ***Line 309-312***  *These findings suggest that there is a correlation between pain, functional status, and quality of life among Jordanian women with breast cancer. Specifically, it indicates that as patients experience more interference in their functional status, their quality of life declines.*  ***Line 315-326***  *In addition to the correlation analysis, a hierarchical linear regression analysis was conducted to measure the impact of pain severity (independent variable) on the quality of life of cancer patients (dependent variable) while considering the influence of cancer stage. Furthermore, this analysis was employed to manage other factors, particularly the cancer stage effectively.*  *The result indicates that the control variable, breast cancer stage, accounts for 2.2% of the variability in the quality of life among Jordanian women diagnosed with breast cancer. In addition, the severity of pain accounts for 3.4% of the differences in quality of life experienced by women diagnosed with breast cancer in Jordan. In addition, the P-value for the independent variable, pain severity (0.023), is below the 5% significance level. The data shows that there is a statistically significant relationship between the severity of pain and the quality of life among Jordanian women with breast cancer, as indicated by a beta value of -0.036. (Table 12).* | ^<https://www.google.com/imgres?imgurl=http://www.maltaxterra.com/wp-content/uploads/2015/03/big_tick.png&imgrefurl=http://www.maltaxterra.com/race-entry-list-end-february/&docid=BiOg4ILiGrs_mM&tbnid=bF9PzfQ9lblDFM:&vet=1&w=256&h=256&bih=652&biw=1366&ved=0ahUKEwiW9pvutI3XAhUMEVAKHYoxAzsQxiAIHSgF&iact=c&ictx=1>^ |
| **4.**  **4.1.**  **4.2.** | A mismatch was seen in the results section between the narrative and tables 3, **4**,  and 9. The results described in the text are different from those in these tables. | The sequence (Low, Moderate, High) in Table 3 is corrected.  The narrative was corrected to be consistent with table 4  The sequence (Low, Moderate, High) in Table 9 is corrected | ***Line 251-254*** *Notably, 88% of the respondents have a low level of pain severity, 6.7% have a moderate level of pain severity, and 5.3% have a high level of pain severity (Table 3).* ***Table 3: Summary statistics for pain severity.*** ***Pain Severity*** \| ***N*** \| ***%***; *Low* \| *132* \| *88.0*; *Moderate* \| *10* \| *6.7*; *High* \| *8* \| *5.3*; *Total* \| *150* \| *100.0* ***Line 257-258*** *Also, 84.7% of the respondents have a high pain Interference Score, 14.7% have a moderate pain interference score, and 0.7% have a low pain Interference Score (Table 4).* ***Table 4: Summary statistics for pain interference score.*** ***Pain Interference Score*** \| ***N*** \| ***%***; *Low* \| *1* \| *0.7*; *Moderate* \| *22* \| *14.7*; *High* \| *127* \| *84.7*; *Total* \| *150* \| *100.0* ***Line 287-288*** *35.3% of the respondents have a low quality of life, 32.7% have a moderate quality of life, and 32% have a high quality of life (Table 9).* ***Table 9: Summary statistics for quality of life ( N=150).*** ***Quality Of Life Index*** \| ***N*** \| ***%***; *Low* \| *53* \| *35.3*; *Moderate* \| *49* \| *32.7*; *High* \| *48* \| *32.0*; *Total* \| *150* \| *100.0* | ^<https://www.google.com/imgres?imgurl=http://www.maltaxterra.com/wp-content/uploads/2015/03/big_tick.png&imgrefurl=http://www.maltaxterra.com/race-entry-list-end-february/&docid=BiOg4ILiGrs_mM&tbnid=bF9PzfQ9lblDFM:&vet=1&w=256&h=256&bih=652&biw=1366&ved=0ahUKEwiW9pvutI3XAhUMEVAKHYoxAzsQxiAIHSgF&iact=c&ictx=1>^ |
| **5.** | P values are better reported with the statistics where applicable. For example, the correlation matrix in Table 11, p-values obtained can be more convincing if they were included in the table with the r statistic. | Explicitly stated | ***Line 314***  **. Correlation is significant at 0.01 *[p value]* (2-tailed). | ^<https://www.google.com/imgres?imgurl=http://www.maltaxterra.com/wp-content/uploads/2015/03/big_tick.png&imgrefurl=http://www.maltaxterra.com/race-entry-list-end-february/&docid=BiOg4ILiGrs_mM&tbnid=bF9PzfQ9lblDFM:&vet=1&w=256&h=256&bih=652&biw=1366&ved=0ahUKEwiW9pvutI3XAhUMEVAKHYoxAzsQxiAIHSgF&iact=c&ictx=1>^ |
|  | **Reviewer #2:** |  |  |  |
| **1.** | Literature Review: The literature review section could be expanded to include more recent studies and a broader range of perspectives on the topic. Incorporating more international research would provide a better context for the findings. | Two paragraphs were added to the literature review | ***From lines 91 to 113*** | ^<https://www.google.com/imgres?imgurl=http://www.maltaxterra.com/wp-content/uploads/2015/03/big_tick.png&imgrefurl=http://www.maltaxterra.com/race-entry-list-end-february/&docid=BiOg4ILiGrs_mM&tbnid=bF9PzfQ9lblDFM:&vet=1&w=256&h=256&bih=652&biw=1366&ved=0ahUKEwiW9pvutI3XAhUMEVAKHYoxAzsQxiAIHSgF&iact=c&ictx=1>^ |
| **2.** | Clarity of Sentences: Line 83 contains sentences that are unclear and require further clarification for better understanding. | Rewritten to be more clear | ***Line 69-71***  *Physicians generally have higher perceptions of health and well-being compared to patients, particularly among younger patients and those experiencing severe symptoms.* |  |
| **3.** | Settings and Sampling: There is a discrepancy regarding the settings from which the data were collected. While the manuscript mentions that information was collected from public, military, private, and nonprofit sectors, the sample and sampling section only references Al-Bashir Governmental Hospital. This needs to be clarified. Additionally, please specify the number of patients selected from each setting in Table 1. | Data was collected **only from Al-Bashir Hospital.**  Under the setting is an introductory sentence about the sittings offering general health care for oncology patients in Jordan. |  | ^<https://www.google.com/imgres?imgurl=http://www.maltaxterra.com/wp-content/uploads/2015/03/big_tick.png&imgrefurl=http://www.maltaxterra.com/race-entry-list-end-february/&docid=BiOg4ILiGrs_mM&tbnid=bF9PzfQ9lblDFM:&vet=1&w=256&h=256&bih=652&biw=1366&ved=0ahUKEwiW9pvutI3XAhUMEVAKHYoxAzsQxiAIHSgF&iact=c&ictx=1>^ |
| **4.** | Sample Size Calculation: Include a citation for the descriptive study formula used to calculate the sample size. | Included | ***Line 146-156*** | ^<https://www.google.com/imgres?imgurl=http://www.maltaxterra.com/wp-content/uploads/2015/03/big_tick.png&imgrefurl=http://www.maltaxterra.com/race-entry-list-end-february/&docid=BiOg4ILiGrs_mM&tbnid=bF9PzfQ9lblDFM:&vet=1&w=256&h=256&bih=652&biw=1366&ved=0ahUKEwiW9pvutI3XAhUMEVAKHYoxAzsQxiAIHSgF&iact=c&ictx=1>^ |
| **5.**  **5.1.**  **5.2.** | Data Analysis: The data analysis section would benefit from a more detailed explanation of the statistical methods used.  Clarifying how the authors handled potential confounding variables would add to the study's credibility.  Additionally, consider analyzing whether there are differences between groups from different settings regarding the study variables. | A little pet explanation of the Pearson correlation test was added.  Confounding variables such as cancer stage were controlled statistically using the technique of "regression." clarification was added.  Data collected only from one sitting | ***Line 224-232***  *The Pearson correlation test is a statistical method for calculating the correlation coefficient between variables. The values vary from 1 to -1. A value of 0 indicates a lack of correlation between the variables.*  *A positive result indicates a direct relationship, while a negative result indicates an inverse relationship (Nettleton, 2014). Correlation is a measure of the strength of the relationship between two variables. It can be described using the guidelines proposed by Evans (1996) based on the absolute value of (r): 0.00 to 0.19 is considered "very weak," 0.20 to 0.39 is considered "weak," 0.40 to 0.59 is considered "moderate," 0.60 to 0.79 is considered "strong," and 0.80 to 1.00 is considered "very strong."*  ***Line 233-236***  *The statistical technique of "regression" was utilized to manage confounding variables, such as cancer stage. The method aims to assess whether the variables of interest have a statistically significant impact on the dependent variable, which is the quality of life, while considering other variables, precisely the cancer stage.*  *--* |  |
| **6.**  **6.1.**  **6.2.** | o Table 1: Include the number of patients selected from the various settings (governmental hospital, military, and other settings).  o Table 4: There is a contradiction between Line 220 and the data presented in the table regarding the percentage of respondents with low pain interference scores. This needs to be corrected.  o Line 227: Correct the phrase "with arrange" to ensure clarity. | Data collected only from one sitting  Corrected  Corrected | ***Line 257-258***  *Also, 84.7% of the respondents have a high pain Interference Score, 14.7% have a moderate pain interference score, and 0.7% have a low pain Interference Score.*  ***Line 263***  *with a range between* | ^<https://www.google.com/imgres?imgurl=http://www.maltaxterra.com/wp-content/uploads/2015/03/big_tick.png&imgrefurl=http://www.maltaxterra.com/race-entry-list-end-february/&docid=BiOg4ILiGrs_mM&tbnid=bF9PzfQ9lblDFM:&vet=1&w=256&h=256&bih=652&biw=1366&ved=0ahUKEwiW9pvutI3XAhUMEVAKHYoxAzsQxiAIHSgF&iact=c&ictx=1>^ |
| **7.** | Discussion and Implications: The discussion could be enriched by providing a more in-depth analysis of the implications of the findings for clinical practice and policy. | Section rewritten | ***Line 418-435*** |  |
| **8.**  **8.1.** | Formatting and Clarity: There are minor issues with the manuscript's formatting and clarity. For instance, some tables and figures could be better formatted to enhance readability.  Additionally, ensuring consistent use of terminology throughout the manuscript would improve its overall coherence. | Checked and Corrected  Checked for terminology use consistency | *All over the manuscript*  *All over the manuscript* |  |
